# SARIFA Is Associated with Lymph Node Metastases in PT3 and PT4 Gastric Cancers

**DOI:** 10.3390/cancers17213593

**Published:** 2025-11-06

**Authors:** Krešimir Mustapić, Petar Đolonga, Tomislav Ivanović, Ana Paparella Karaman, Luka Minarik, Katarina Vukojević, Merica Glavina Durdov

**Affiliations:** 1 Department of Surgery, University Hospital of Split, 21000 Split, Croatia; kresimir.mustapic@gmail.com (K.M.); tomo.mefst@gmail.com (T.I.); 2 Department of Pathology, Forensic Medicine and Cytology, University Hospital of Split, 21000 Split, Croatia; pdolonga@kbsplit.hr (P.Đ.); anapaparella@hotmail.com (A.P.K.); 3 Department of ENT, County Hospital of Čakovec, 40000 Cakovec, Croatia; luka.minarik@mefst.hr; 4 Department of Anatomy, Histology and Embryology, University of Split School of Medicine, 21000 Split, Croatia; katarina.vukojevic@mefst.hr

**Keywords:** gastric cancer, SARIFA, prognostic markers

## Abstract

**Simple Summary:**

Advanced gastric cancer is generally associated with a poor prognosis. Stroma AReactive Invasive Front Area (SARIFA) is a recently recognized aggressive histological feature, defined as five tumor cells in direct contact with adipocytes within perigastric, submucosal, or perivascular adipose tissue. The aim of our retrospective study was to evaluate the correlation of SARIFA with pathohistological variables and its impact on overall survival. A cohort of 102 Croatian patients with locally advanced gastric cancer was analyzed, and a significant association between SARIFA and nodal metastases as well as perineural invasion was observed. Patients with both lymphovascular invasion and SARIFA had a significantly higher proportion of affected lymph nodes. They also exhibited a shorter, though not statistically significant, overall survival compared with patients with one or neither of these factors (median 9.2 vs. 16.1 months). A positive SARIFA status may serve as a biomarker of invasiveness and an additional prognostic risk factor.

**Abstract:**

**Background/Objectives**: Advanced gastric cancer usually has an unfavorable prognosis. Stroma AReactive Invasion Front Area (SARIFA) is a newly recognized biomarker of aggressiveness, easily recognized as five tumor cells in direct contact with adipocytes in perigastric, submucosal, and perivascular adipose tissue. We investigated this phenomenon and correlated it with other pathohistological variables. **Material and Methods**: The sample includes 102 Croatian patients with locally advanced gastric cancer, who underwent total gastrectomy/lymphadenectomy between 2012–2018 and in 2023 at University Hospital Split, Croatia, and had pathological stage pT3 or pT4. Representative histological specimens were analyzed for SARIFA, and results were compared with other variables and overall survival. External validation and gene expression analysis of CD36 and FABP4 were performed using the TCGA-STAD cohort. **Results**: SARIFA was significantly associated with positive pN status (*p* = 0.009) and perineural invasion (*p* = 0.043). Patients with SARIFA had a more than fivefold increased risk of nodal involvement (OR = 6.35; 95% CI: 1.35–29.84; *p* = 0.019). Lymphovascular invasion (LVI) was associated with nodal disease (OR = 4.39; 95% CI: 1.194–16.143; *p* = 0.026), and SARIFA was marginally associated (OR = 4.886; 95% CI: 0.985–24.241; *p* = 0.052). Patients who had both LVI and SARIFA had a higher proportion of affected lymph nodes (*p* = 0.009). SARIFA status did not significantly affect overall survival. Gene expression analysis showed a significant increase in CD36 expression, while FABP4 expression was elevated but not statistically significant, in SARIFA-positive cases. **Conclusions**: SARIFA could be used as a marker for invasiveness and further investigated due to its predictive potential.

## 1. Introduction

According to GLOBOCAN 2022, gastric cancer affects approximately 136,000 people in Europe annually and accounts for more than 95,000 deaths [1]. The current gold standard for treating clinical stages 3 and 4 is gastrectomy followed by postoperative chemotherapy and/or immunotherapy [2]. Today, gastric adenocarcinoma is classified into four molecular subgroups named Epstein–Barr virus-positive (EBV+), microsatellite instable (MSI), genomically stable (GS), and chromosomally unstable (CIN). However, genetic analysis is not performed in routine clinical practice [3]. It is well known that lymphovascular invasion (LVI), perineural invasion (PNI), and advanced pathological stage (pTNM) correlate with poorer survival outcomes [4,5,6]. Apart from traditional histological parameters, recent evidence suggests that invasion front biomarkers like tumor budding [7], tumor deposits [8], and tumor–stroma ratio [9] could be useful for improved risk stratification and prognosis prediction in patients with gastric cancer. Recently, German authors described a novel adverse biomarker on the invasive front, SARIFA (Stroma AReactive Invasion Front Area). SARIFA is defined as small clusters of tumor cells in direct contact with adipocytes, without intervening connective tissue stroma or inflammatory response [10]. SARIFA can be analyzed in the invasion tumor front in submucosal, perivascular, perineural, and perigastric adipose tissue [11].

Over the last decade, tumor biology research has increasingly focused on the role of obesity in tumor pathophysiology, with evidence suggesting that adipocytes, through energy supply and regulation of autophagy, may promote tumor growth and metastasis [12,13,14]. Recent studies by German researchers who initially described SARIFA suggest that direct contact between tumor cells and adipocytes may enable tumor cells to exploit fatty acid metabolism, thereby facilitating further tumor progression in gastric cancer [15,16]. Duong et al. demonstrated that co-culture of breast cancer cells with adipocytes leads to a reduction in the number and size of lipid droplets within adipocytes. Histologically, adipocytes at the invasive front of the tumor appear smaller and depleted of lipids compared with those distant from the tumor [17].

Two key proteins involved in this process that are upregulated in cancer cells in close proximity to adipocytes are CD36 and fatty acid-binding protein 4 (FABP4). During malignant transformation, cancer cells undergo lipidomic remodeling and express the transport protein CD36, a fatty acid translocase that enables tumor cells to uptake fatty acids from adjacent adipocytes [18]. FABP4 is involved in the intracellular transport and metabolism of fatty acids within cancer cells [19].

In this single-center study from Croatia, we analyzed the presence of SARIFA in surgically resected, locally advanced gastric cancer and examined its association with other, well-established pathohistological hallmarks of tumor aggressiveness and overall survival. Because our initial cohort was relatively small and ethnically homogeneous, we further validated our findings using the TCGA STAD dataset.

## 2. Materials and Methods

### 2.1. Sample Size and Data Collection

This study analyzed 102 patient specimens from total or partial gastrectomy with regional lymphadenectomy performed at the Department of Surgery, Division of Abdominal Surgery, University Hospital of Split, Croatia, during 2012–2018 and 2023. The interruption between 2019 and 2022 was caused mainly by the COVID pandemic, which drastically reduced the surgical program. The main inclusion criterion was a high pathological tumor stage, classified as cancer that invades adventitia (pT3 stage) or cancer that invades neighboring structures (pT4 stage) according to the AJCC 8th edition pTNM classification [9]. Basic demographic data (age and sex) and clinicopathological information were obtained from the archives of the Department of Pathology, Forensic Medicine, and Cytology. The follow-up period was defined as the time interval, expressed in months, from the date of surgery until death or the end of follow-up on June 30, 2025. The collected clinicopathological variables included pathological tumor grade (pT3 or pT4), lymph node involvement (patients with at least one infiltrated lymph node were classified as positive), Lauren histological subtype (intestinal or diffuse/mixed), and the presence or absence of LVI and PNI. For each patient, the proportion of involved lymph nodes was calculated as the number of infiltrated lymph nodes divided by the total number of isolated regional lymph nodes.

### 2.2. SARIFA Status Assessment

Representative paraffin-embedded tumor tissue blocks were retrieved from the archives of the Department of Pathology, Forensic Medicine and Cytology, University Hospital of Split, Croatia. Sections were cut at a thickness of three µm and stained with hematoxylin and eosin (H&E). Two pathologists (MGD and PĐ) independently examined a single slide from each patient, specifically the one showing the deepest tumor invasion, to assess the presence of the SARIFA phenomenon. SARIFA was defined as direct contact between tumor cells and adipocytes at the invasive tumor front within the perigastric fat or perivascular tissue, without intervening connective tissue and/or inflammatory cells [9,10]. SARIFA-positivity was defined as the presence of at least one focus comprising five or more tumor cells in direct contact with adipocytes, confirmed by both pathologists. Interobserver agreement for the SARIFA assessment was substantial, with a Cohen’s kappa of 0.701 (95% CI: 0.566–0.836). All slides on which the SARIFA status differed between pathologists were resolved by consensus review.

### 2.3. TCGA Cohort

Clinical, pathological, and mRNA expression data for 108 patients with locally advanced gastric cancer were retrieved from the publicly available cBioPortal for Cancer Genomics database (https://cbioportal.org/, “Stomach Adenocarcinoma TCGA, PanCancer Atlas database”, accessed on 25 October 2025). We accessed the digital slides via https://cancer.digitalslidearchive.org (accessed on 23 October 2025). We analyzed the correlation between the presence of SARIFA and clinical, pathological, and molecular parameters, including CD36 and FABP4 mRNA expression. The inclusion criterion for histologic assessment was the presence of perigastric or perivascular adipocytes at the invasive margin, as previously described.

### 2.4. Statistical Analysis

Categorical data are presented as absolute frequencies (*n*) and percentages (%). Differences between categorical variables were determined using the chi-square test, Fisher’s exact test, and the Wilcoxon test where applicable. For numerical data, the normality of distribution was tested using the Shapiro–Wilk test. Due to significant deviation from normality, numerical data were expressed as median and interquartile range (IQR). Significance of differences between groups was established using the Mann–Whitney U test. Binary logistic regression was used to evaluate the association between histological adverse factors and lymph node metastases. Gene set enrichment analysis was performed using the Hallmark (H) gene sets obtained from the Molecular Signatures Database (MSigDB) via the msigdbr R package v25.1.1, and enrichment scores were calculated using fgsea. Survival analysis was performed by the Kaplan–Meier method. A *p*-value of <0.05 was considered statistically significant. All statistical analyses were performed using IBM SPSS Statistics for Windows, Version 26 (IBM Corp., Armonk, NY, USA), and RStudio software (v. 2024.12.1+563).

## 3. Results

### 3.1. Clinicopathological Correlates of SARIFA in the Institutional Cohort

**Figure 1:**
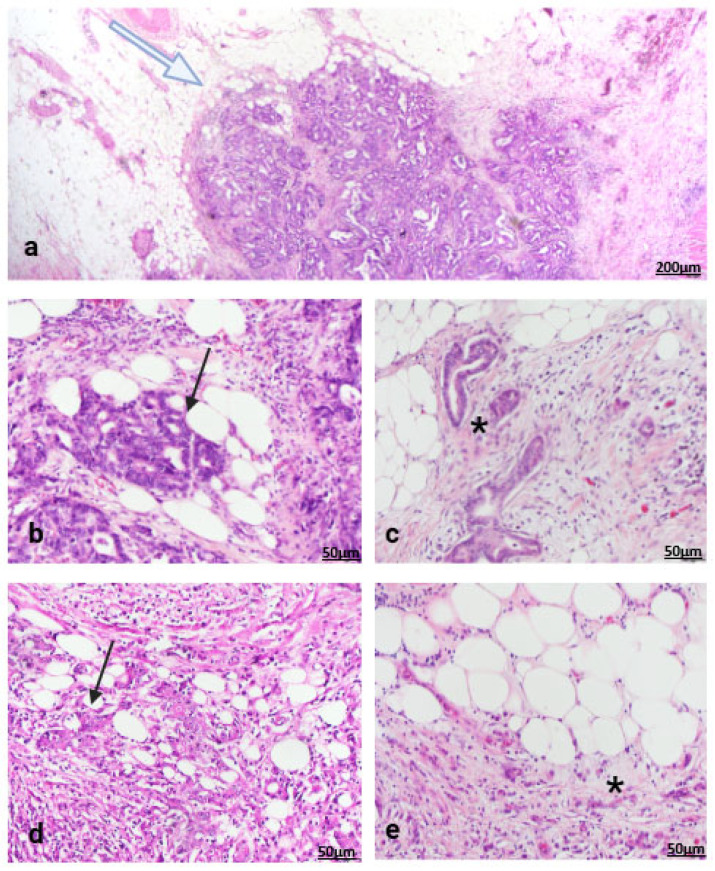
At the invasive front of gastric cancer (**a**), (white arrow), SARIFA is defined as direct contact between at least five tumor cells and adipocytes (black arrows); H&E, scale bar 200 µm. In SARIFA-negative cases, fibroblasts or inflammatory cells (asterisk) are interposed between cancer cells and adipocytes. Representative images show intestinal subtype gastric cancer positive (**b**) and negative (**c**) for SARIFA, and diffuse subtype gastric cancer positive (**d**) and negative (**e**) for SARIFA; H&E, scale bar 50 µm.

SARIFA status was evaluated in 102 gastric cancer specimens in pathological stages 3 or 4, out of which 44.1% (*n* = 45) exhibited at least one SARIFA focus. SARIFA was assessed in standard HE sections in both histological subtypes of gastric cancer (Figure 1).

Among SARIFA-positive patients, 95.6% had regional lymph node metastases, compared to 77.2% of SARIFA-negative patients. This difference was statistically significant (*p* = 0.009). Other adverse pathohistological features were more frequently observed in SARIFA-positive patients; however, only PNI showed a statistically significant association (*p* = 0.043) (Table 1).

Univariate logistic regression was used to evaluate whether key clinicopathological features are associated with nodal involvement in gastric cancer. As expected, LVI and PNI were associated with a higher risk of nodal metastases (OR = 6.05; 95% CI: 1.83–19.98; *p* = 0.003 and OR = 3.18; 95% CI: 1.04–9.75; *p* = 0.043, respectively). SARIFA presence was also a significant predictor, with patients exhibiting SARIFA having more than a fivefold increased risk of nodal involvement (OR = 6.35; 95% CI: 1.35–29.84; *p* = 0.019), while other tested variables were not significantly associated with nodal metastases (Table 2).

A multivariate logistic regression model with variables that were significantly associated with a higher risk of nodal involvement in univariate analysis was used to assess independent predictors of lymph node metastases. The model was statistically significant, χ^2^ (3) = 14.815, *p* = 0.002, and explained 23.9% of the variance in lymph node involvement (Nagelkerke R^2^), correctly classifying 85% of cases. As expected, LVI remained strongly associated with nodal disease (OR = 4.39; 95% CI: 1.194–16.143; *p* = 0.026). SARIFA presence was marginally associated with lymph node involvement (OR = 4.886; 95% CI: 0.985–24.241; *p* = 0.052). In contrast, PNI was not independently associated with lymph node metastases in the multivariate model (Table 3).

**Figure 2:**
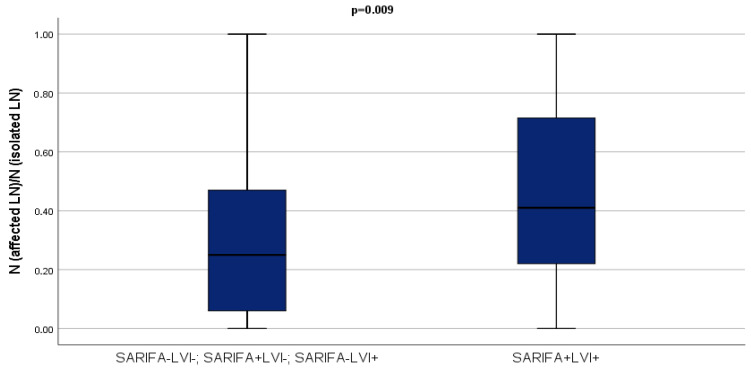
Proportion of affected lymph nodes in the SARIFA+LVI+ group compared to the other SARIFA/LVI groups (Mann–Whitney U test).

Considering the biological overlap between SARIFA and LVI, and the marginal influence of SARIFA in the multivariate analysis, SARIFA status and LVI presence were combined into a single variable. This approach aimed to assess whether the simultaneous presence of both adverse pathological features could better stratify the risk of nodal involvement than either factor alone. Patients who had both SARIFA and LVI present in their specimens had a greater tendency to have a higher proportion of affected lymph nodes (*p* = 0.009) (Figure 2).

To assess whether the combined presence of SARIFA and LVI better predicts lymph node metastases, a multivariate regression analysis was performed, including the combined SARIFA-LVI variable and PNI. The model was statistically significant (χ^2^ (2) = 12.040, *p* = 0.002), explaining 19.7% of variance in lymph node involvement and correctly classifying 85.3% of cases. Combined SARIFA and LVI presence increased the risk of nodal metastases nearly tenfold (OR: 9.913; 95% CI: 1.232–79.735; *p* = 0.031). PNI was not a significant predictor in this model either (Table 4).

**Figure 3:**
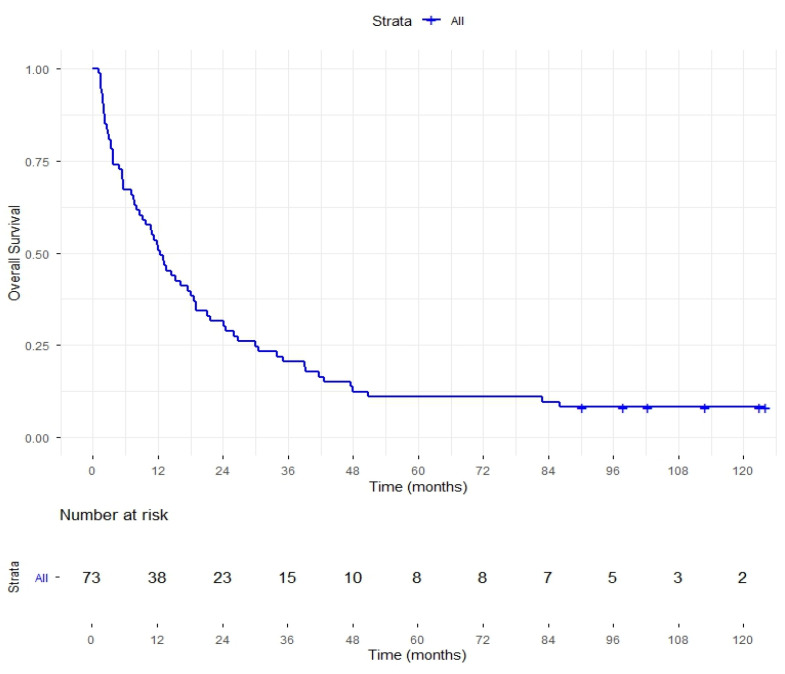
Kaplan–Meier cum survival for 73 patients followed from the date of surgery until death or the end of follow-up on 30 June 2025, expressed in months.

**Figure 4:**
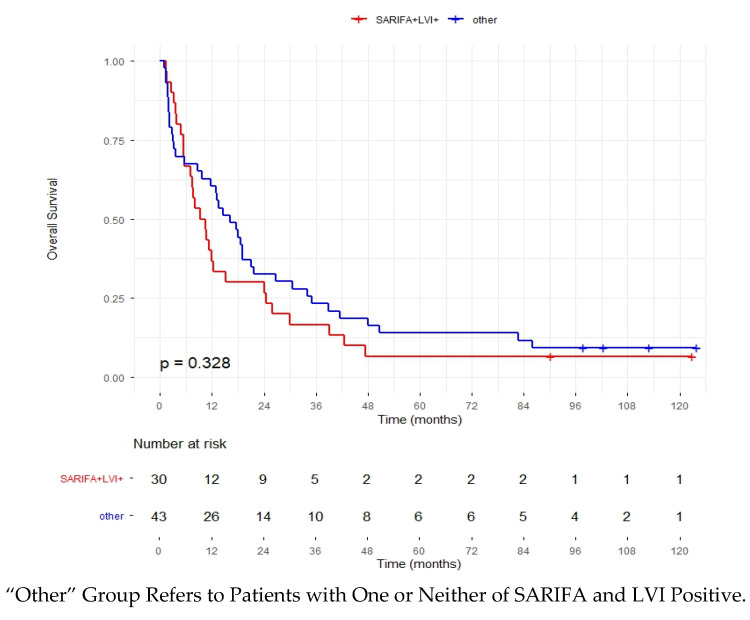
Kaplan–Meier cum Survival According to Presence of LVI and SARIFA. The difference between groups was assessed using the log-rank test.

Kaplan–Meier survival analysis was conducted in 73 patients diagnosed between 2012 and 2018, with follow-up from diagnosis to death or censoring at June 2025. Of the 73 patients who were followed, 6 (8.2%) were censored. The median survival time was 12.4 months (95% CI: 8.77–16.02). Estimated survival values at key time points were 1 year—50.7% (95% CI: 40.4–63.6); 2 years—30.5% (95% CI: 22.5–44.2); and 5 years—approximately 10.6% (95% CI: 5.7–21.1), with values for 2 and 5 years determined by interpolation between two-time steps in the survival table (Figure 3). Among the patients who were followed, survival analysis was performed within two subgroups defined as SARIFA+LVI+ (*n* = 30; 41.1%) and “other”, in which one or neither of these variables was positive (*n* = 43; 58.9%). In the SARIFA+LVI+ group, the median survival time was 9.2 months (95% CI: 4.91–13.49), and the estimated survival time at key time points was 1 year—36.7% (95% CI: 22.9–58.7), 2 years—27.8% (95% CI: 17.4–51.8) and 5 years—6.7% (95% CI: 1.7–25.4). In the “other” group, the median survival time was 16.1 months (95% CI: 9.8–22.4), with an estimated survival rate at 1 year of 60.1% (95% CI: 47.5–76.9), at 2 years of 31.5% (95% CI: 21.2–50.1) and at 5 years of 13.3% (95% CI: 6.6–29.3). There was no statistically significant difference in overall survival between the SARIFA+LVI+ group and the “other” group (log-rank test, ꭓ^2^ = 0.955; *p* = 0.328) (Figure 4).

### 3.2. Clinicopathological Correlates of SARIFA in the TCGA-STAD Cohort

We used the publicly available TCGA cohort to assess whether there was a clinical or biological association between SARIFA status and pathological markers, as well as CD36 and FABP4 mRNA expression. 108 consecutive cases of locally advanced gastric cancer (pT3 and pT4) were selected. The inclusion criterion for histological evaluation was the presence of perigastric or perivascular adipocytes at the invasive margin to determine SARIFA. Demographic and clinicopathological data of these patients were compared according to SARIFA status (Figures S1 and S2). SARIFA-positive patients tended to have higher pN stages (32% of SARIFA-positive patients had pN3 disease compared with 13% of SARIFA-negative patients), although this difference was not statistically significant (*p* = 0.233). There was no association between the presence of SARIFA and the occurrence of distant metastases. No statistically significant differences in SARIFA status were observed in relation to the molecular tumor profile. SARIFA status was not associated with differences in overall survival (OS) or progression-free survival (PFS) in patients from the TCGA cohort (Figure S3).

### 3.3. Validation of SARIFA-Related Expression of CD36 and FABP4 in the TCGA STAD Dataset

**Figure 5:**
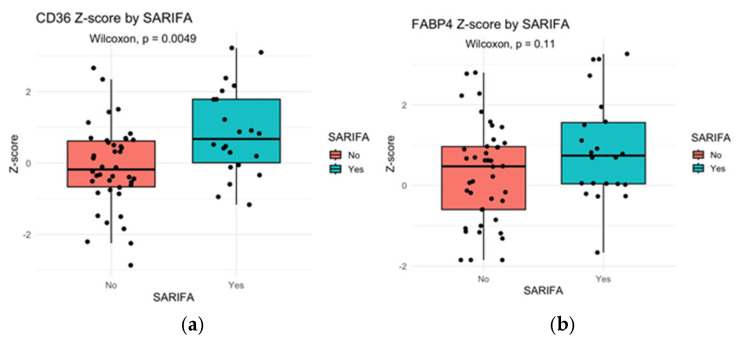
Box plots comparing the expression of CD36 (**a**) and FABP4 (**b**), two genes implicated in lipid metabolism. CD36 was significantly overexpressed in the SARIFA-positive group (*p* = 0.0049). In contrast, FABP4 showed slightly higher expression in the SARIFA-positive cohort, but this difference did not reach statistical significance (*p* = 0.11).

CD36 showed significantly higher expression in SARIFA-positive gastric cancers (*p* = 0.0049, Wilcoxon test). FABP4, another gene reported to be associated with SARIFA, was expressed 1.56 times higher in SARIFA-positive gastric cancers, but this difference did not reach statistical significance (*p* = 0.11, Wilcoxon test) (Figure 5).

There were no differences in OS and PFS in relation to CD36 and FABP4 expression (Figure S4).

## 4. Discussion

In this retrospective study involving 102 patients with regionally advanced gastric cancer (pT3 and pT4), we evaluated the prognostic significance of Stroma AReactive Invasion Front Area (SARIFA), a novel histopathological biomarker. To assess the nature of intercellular interactions in SARIFA areas, we analyzed the expression of CD36 and FABP4, genes known to be involved in alterations of fatty acid metabolism at the interface between tumor cells and adipocytes [18,19]. Our findings suggest the potential of SARIFA as a predictor of tumor aggressiveness, particularly when it appears alongside lymphovascular invasion (LVI).

SARIFA was initially described in colorectal cancer as the direct contact between tumor cell clusters (of at least 5 cells) and adjacent adipocytes at the invasive front, without any intervening connective stroma or inflammatory infiltrates [10]. Its application has since expanded to other malignancies, including gastric, pancreatic, and prostate cancers, where it is associated with worse prognosis, altered immune microenvironments, and metabolic reprogramming [15,16,17,18,20].

In our study, SARIFA positivity was observed at almost double the rate reported by Grosser et al. (40% vs. 20%, respectively) [16]. This discrepancy may be due to our focus on locally advanced gastric cancer or differences in the distribution of histological subtypes. In our sample, SARIFA positivity was more often observed among diffuse and mixed-type cancers, known for their infiltrative growth patterns, compared to intestinal-type cancers. Contrastingly, Ulase et al. reported opposing findings, underscoring the need for further detailed investigations [21]. In addition, our SARIFA-positive tumors were associated with adverse histological features, particularly PNI, and showed a non-significant increase in LVI. These observations align with the hypothesis that the absence of stromal or inflammatory barriers in SARIFA regions may facilitate direct invasion into neural and vascular structures [22].

In our cohort, lymph node metastases were more frequent in SARIFA-positive patients. In univariate logistic regression, SARIFA positivity was associated with more than a six-fold increase in the likelihood of nodal involvement. In the multivariate model, the presence of SARIFA showed a trend toward significance in its association with lymph node involvement (OR = 4.886; 95% CI: 0.985–24.241; *p* = 0.052). Similarly, in our analysis of the TCGA STAD cohort, SARIFA was slightly more frequent in patients with pN3, although the difference among pN groups was not statistically significant. Kuhn et al. highlighted that LVI in breast cancer is typically located in peritumoral areas within 1 mm of the invasive carcinoma [23]. This suggests that both LVI and SARIFA are generally observed at the invasive front of tumors. We further propose that LVI may occur more frequently in regions adjacent to SARIFA, where the stromal response is lacking, thereby increasing the likelihood of contact between tumor cells and the vasculature of loose connective and adipose tissue. The marginal association of SARIFA with lymph node metastases may reflect this pattern, but further investigation in a larger cohort is needed to draw definitive conclusions, as the small sample size combined with weak to moderate collinearity in our dataset may limit the presented results. Although this association was marginally non-significant in multivariate analysis, combining SARIFA with LVI in a composite variable improved predictive accuracy. Patients who were positive for both biomarkers exhibited a tenfold increased risk of nodal metastases and had a significantly higher proportion of affected lymph nodes.

These findings are biologically plausible, given prior molecular studies indicating a shift in lipid metabolism in SARIFA-positive tumors.

Reitsam et al. demonstrated upregulation of fatty acid transport genes such as CD36 in SARIFA-positive colorectal tumors, suggesting that lipid exchange between tumors and adipocytes plays a role in invasion [24]. CD36, also known as fatty acid translocase (FAT), is a multifunctional membrane protein that mediates the uptake of long-chain fatty acids into cells. It is expressed on various cell types, including adipocytes, macrophages, and endothelial cells [25]. Its expression on tumor cells facilitates the uptake of free fatty acids, contributing to lipid metabolic reprogramming, increased proliferation, and invasiveness of tumors [26]. Similarly, gene expression analysis performed on the TCGA-STAD cohort showed overexpression of CD36 in SARIFA-positive cases, indicating fatty acid metabolism changes in SARIFA areas, which is consistent with previous findings in gastric cancer [16]. FABP4 is a cytosolic protein primarily expressed in adipocytes and macrophages. While FABP4 mediates the uptake of free fatty acids [27], our analysis did not show significantly higher expression of this gene in SARIFA-positive gastric cancers. In contrast, Grosser et al. demonstrated that FABP4 expression was observed almost exclusively in SARIFA areas and found a positive correlation between FABP4 expression and the number of macrophages [16]. The immunological characteristics of SARIFA support its pro-metastatic potential. Tapainen et al. reported that SARIFA-positive tumors exhibit reduced infiltration of T-cells and NK-cells, along with an increase in immunosuppressive macrophage populations, which further facilitates unopposed tumor growth into adipose tissue [28,29,30].

SARIFA is an easily recognizable biological phenomenon on routine H&E slides, showing excellent interobserver agreement. It reflects complex intercellular relationships that are yet to be fully elucidated in the context of other negative prognostic biomarkers that may share similar mechanisms, such as tumor budding. In the study by Jakab et al., SARIFA was identified as a negative prognostic factor in esophageal adenocarcinoma, while tumor budding was shown to be a negative predictive factor in squamous cell carcinoma [31]. The mentioned findings suggest that SARIFA may represent a tumor-specific marker; however, it should be further evaluated in larger cohorts and across different tumor types.

This is the first study from Croatia to assess SARIFA in gastric cancer. While the number of publications on SARIFA in gastric cancer remains limited, our findings are consistent with existing data and strengthen the argument for incorporating SARIFA into routine pathological assessments. Its rapid evaluation on standard H&E slides, without the need for immunohistochemistry, offers practical value in clinical workflows.

There are some limitations to our work that should be noted. This was a single-center retrospective study enrolling an ethnically homogeneous population; thus, these observations might not be able to be generalized to the overall or worldwide population. In addition, we cannot stratify our analysis based on confounding factors such as smoking status or alcohol intake. The study’s limited sample size constrains the specificity of the regression model and significantly limits survival analysis, necessitating cautious interpretation.

## 5. Conclusions

These findings underscore the complexity of advanced gastric cancer and highlight the potential prognostic value of SARIFA in conjunction with established histological biomarkers of tumor aggressiveness. Given the paucity of existing data, further research is warranted to elucidate SARIFA’s role in tumor progression and its integration with known prognostic markers.

## Figures and Tables

**Table 1:** Demographic and pathohistological features according to SARIFA status in gastric cancer.

VARIABLE	Total Number of Samples (*n* = 102)	SARIFA Positive (*n* = 45, 44.1%)	SARIFA Negative (*n* = 57, 55.9%)	*p*-Value
Age (years, median, IQR)	75.6 (67–81)	75 (65–79.8)	76 (70–82)	0.354 *
Sex (*n*, %):				0.432 ^#^
Female	36 (35.3)	14 (31.1)	22 (38.6)	
Male	66 (64.7)	31 (68.9)	35 (61.4)	
pT (*n*, %):				0.259 ^#^
pT3	54 (52.9)	21 (46.7)	33 (57.9)	
pT4	48 (47.1)	24 (53.3)	24 (42.1)	
Lymph nodes (*n*, %):				0.009 ^#^
Positive	87 (85.3)	43 (95.6)	44 (77.2)	
Negative	15 (14.7)	2 (4.4)	13 (22.8)	
Proportion of positive lymph nodes (median, IQR)	0.28 (0.09–0.66)	0.33 (0.17–0.7)	0.27 (0.07–0.52)	0.083 *
Lauren histologic type (*n*, %)				0.095 ^#^
Intestinal	48 (47.1)	17 (37.8)	31 (54.4)	
Diffuse/mixed type	54 (52.9)	28 (62.2)	26 (45.6)	
Lymphovascular invasion (*n*, %):				0.124 ^#^
Present	84 (82.4)	40 (88.9)	44 (77.2)	
Not present	18 (17.6)	5 (11.1)	13 (22.8)	
Perineural invasion (*n*, %):				0.043 ^#^
Present	71 (69.6)	36 (80)	35 (61.4)	
Not present	31 (30.4)	9 (20)	22 (38.6)	

*—Mann–Whitney U test; #—chi-squared test; Abbreviations: pT—pathological tumor stage, pT3—tumor invades the adventitia, pT4—tumor invades neighboring structures.

**Table 2:** Univariable logistic regression analysis of demographic and pathohistological factors associated with lymph node metastases in pathological stage 3 and 4 gastric cancer.

VARIABLE	OR (95% CI)	*p*-Value
Age	0.988 (0.933–1.047)	0.690
Sex	1.6 (0.47–5.444)	0.452
pT	1.955 (0.617–6.19)	0.255
Lymphovascular invasion	6.045 (1.829–19.977)	0.003
Perineural invasion	3.18 (1.037–9.754)	0.043
SARIFA status	6.352 (1.352–29.836)	0.019

Abbreviations: pT—pathological tumor stage, SARIFA—Stromal AReactive Invasive Front Area.

**Table 3:** Multivariable logistic regression analysis of pathohistological factors associated with lymph node metastases in pathological stage 3 and 4 gastric cancer.

VARIABLE	OR (95% CI)	*p*-Value
Lymphovascular invasion	4.39 (1.194–16.143)	0.026
Perineural invasion	1.725 (0.487–6.107)	0.398
SARIFA status	4.886 (0.985–24.241)	0.052

Abbreviations: SARIFA—Stromal AReactive Invasive Front Area.

**Table 4:** Final multivariate logistic regression model including the combined SARIFA-LVI variable and PNI in stage 3 and 4 gastric cancer.

VARIABLE	OR (95% CI)	*p*-Value
SARIFA-LVI	9.913 (1.232–79.735)	0.031
PNI	2.532 (0.79–8.113)	0.118

Abbreviations: LVI—lymphovascular invasion, PNI—perineural invasion, SARIFA—Stromal AReactive Invasive Front Area.

## Data Availability

Datasets generated and/or analyzed during the current study are available upon reasonable request from the corresponding author.

## Supplementary Materials

The following supporting information can be downloaded at: https://www.mdpi.com/article/10.3390/cancers17213593/s1, Figure S1: Distribution of patient’s age according to SARIFA status of gastric cancer was not significantly different (*p* = 0.99); Figure S2: Distribution of SARIFA presence based on patient’s gender, cancer molecular subtype, N stage and M stage. Although without statistical significance, SARIFA positive patient cohort is depicted with higher pN stage and male gender. The most common molecular subtype is STAD CIN, while STAD EBV showed a higher proportion in the SARIFA positive group; Figure S3: SARIFA positive patients show an unfavorable outcome on both OS and PFS, although statistically insignificant; Figure S4: Survival analysis of gastric cancer patients based on FABP4 and CD36 mRNA expression. FABP4 and CD36 overexpression show unfavorable OS and PFS.

## Abbreviations

The following abbreviations are used in this manuscript:

LVI	Lymphovascular invasion
PNI	Perineural invasion
SARIFA	Stroma AReactive Invasive Front Area
pTNM	Pathologic tumor node metastasis
pT	Pathological tumor stage
pN	Pathological nodal stage
CI	Confidence interval
OR	Odds ratio
OS	Overall survival
PFS	Progression-free survival
FABP4	Fatty acid-binding protein 4
