# Supplementary material for: SARIFA Is Associated with Lymph Node Metastases in PT3 and PT4 Gastric Cancers
Source: Cancers (Basel). 2025 Nov 6;17(21):3593. doi: 10.3390/cancers17213593 (PMC12609689; doi:10.3390/cancers17213593)
Supplement: Supplementary file 1 [file cancers-17-03593-s001.zip › cancers-3931597-supplementary.pdf]

Supplement

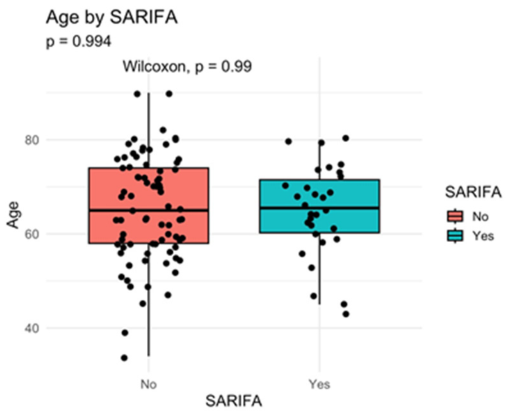

Figure S1. Distribution of patient's age according to SARIFA status of gastric cancer was not significantly different (p=0.99).

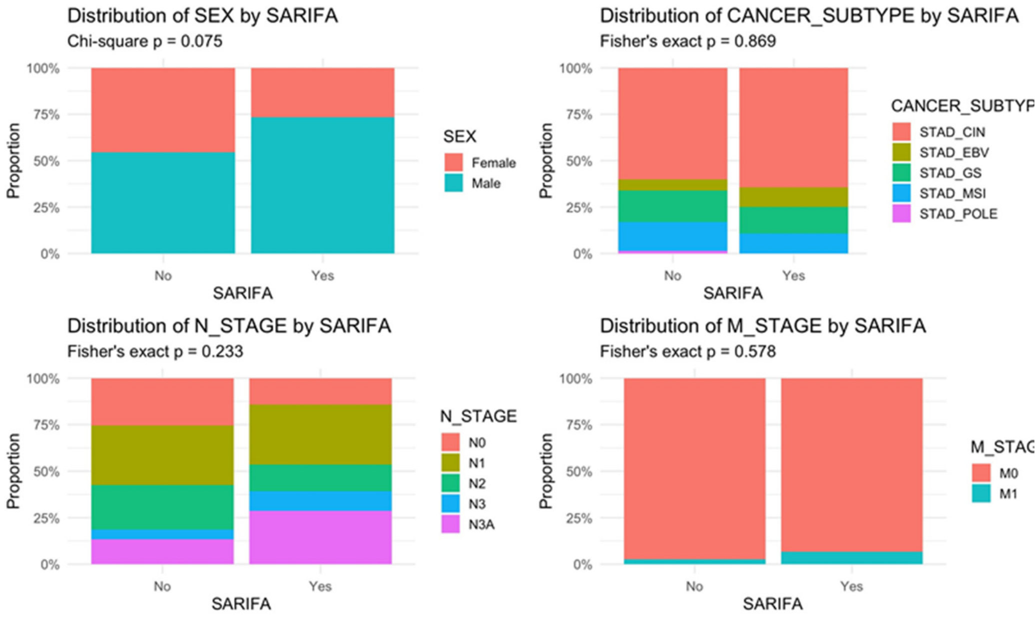

Figure S2. Distribution of SARIFA presence based on patient's gender, cancer molecular subtype, N stage and M stage. Although without statistical significance, SARIFA positive patient cohort is depicted with higher pN stage and male gender. The most common molecular subtype is STAD CIN, while STAD EBV showed a higher proportion in the SARIFA positive group.

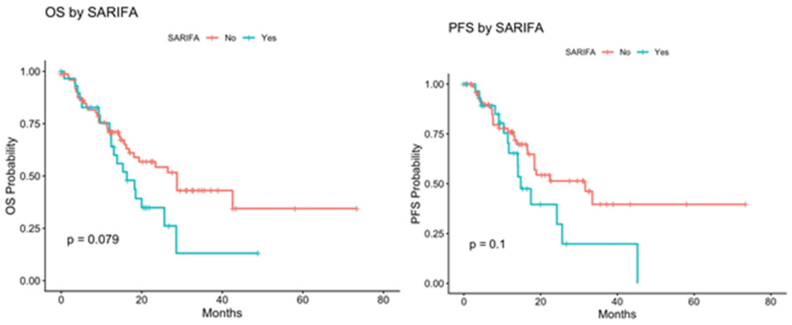

Figure S3. SARIFA positive patients show an unfavorable outcome on both OS and PFS, although statistically insignificant.

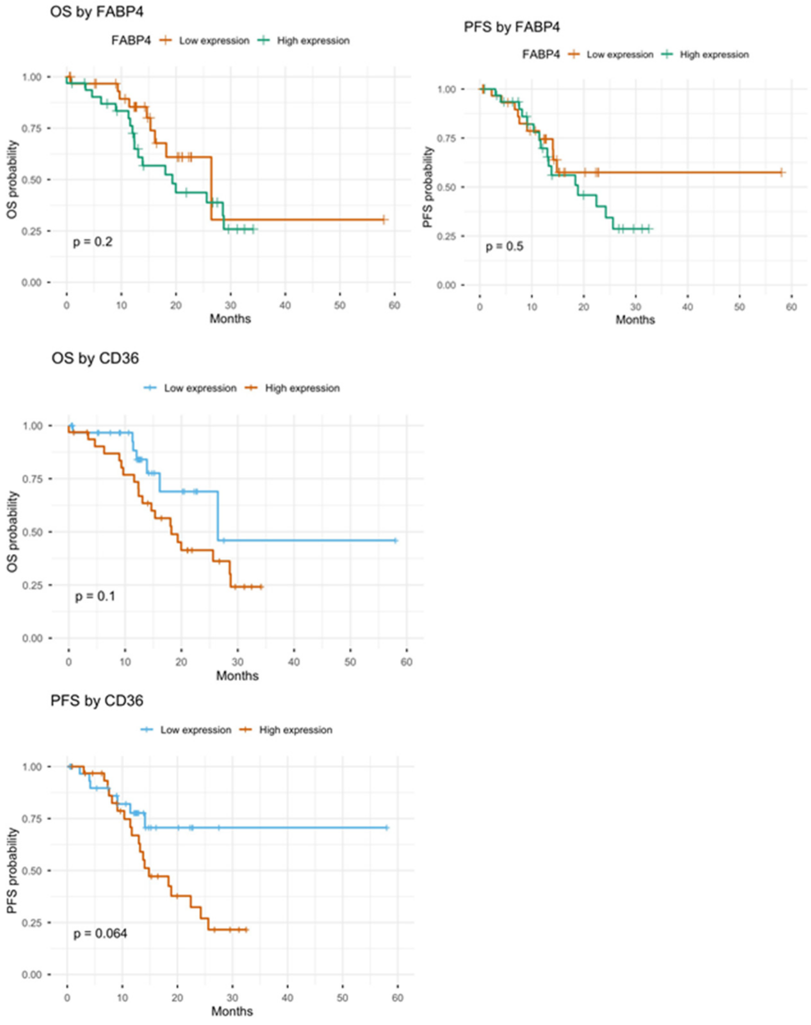

Figure S4. Survival analysis of gastric cancer patients based on FABP4 and CD36 mRNA expression. FABP4 and CD36 overexpression show unfavorable OS and PFS.
